# Bio-Inspired Peptide Membranes for CO_2_ Capture: A Molecular Dynamics Study of A_6_H and A_6_R Interfaces

**DOI:** 10.1021/acs.jpcb.6c00256

**Published:** 2026-04-15

**Authors:** Karinna Mendanha, Guilherme Colherinhas

**Affiliations:** † Instituto de Física, 67824 Universidade Federal de Goiás, Goiânia, Goiás 74690-900, Brazil; ‡ Departamento de Física, CEPAE, 67824 Universidade Federal de Goiás, Goiânia, Goiás 74690-900, Brazil

## Abstract

The increasing concentration of atmospheric CO_2_ demands the development of advanced and sustainable materials for carbon capture. Peptide-based nanostructures have emerged as promising candidates due to their tunable chemistry, biocompatibility, and ability to self-assemble into ordered supramolecular architectures. In this work, we investigate the adsorption behavior of CO_2_ on self-assembled A_6_H and A_6_R peptide membranes through classical molecular dynamics simulations. The A_6_H and A_6_R sequences consist of six alanine residues capped by a terminal histidine or arginine residue, respectively, and self-assemble into stable β-sheet membrane structures whose surface charge distribution and hydration organization are governed by the nature of the terminal residue. After equilibrating the membranes in an aqueous medium, water molecules were removed, and CO_2_ was introduced into the simulation box to evaluate gas–surface interactions under idealized gas-phase contact conditions. The results reveal distinct adsorption mechanisms governed by headgroup chemistry: the imidazole-terminated A_6_H interface exhibits preferential electrostatic and hydrogen-bond-driven interactions with CO_2_, whereas the guanidinium-terminated A_6_R membrane, characterized by a higher surface charge density, promotes enhanced electrostatic attraction and a larger number of CO_2_ binding events. These findings highlight how the chemistry of peptide terminal residues modulates CO_2_ affinity at ordered, self-assembled membrane interfaces, underscoring the potential of bioinspired peptide membranes as tunable platforms for carbon capture. By focusing on experimentally validated supramolecular architectures rather than peptide aggregates or hybrid systems, this study provides molecular-level insights that can inform the rational design of peptide-based sorbent materials for sustainable CO_2_ sequestration.

## Graphical abstract

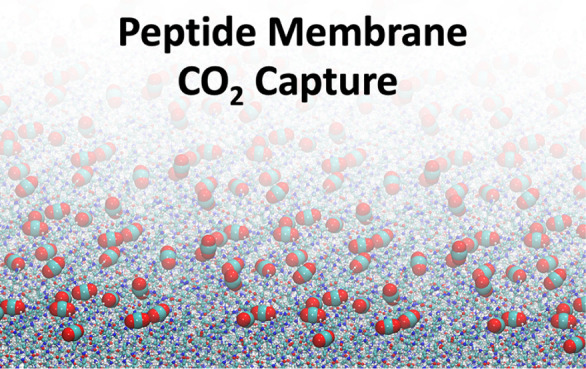

## 1 Introduction

The increasing atmospheric concentration of carbon dioxide (CO_2_) is one of the main causes of global warming, demanding the development of efficient mitigation technologies. Among the available approaches, carbon capture and storage (CCS) and carbon capture, utilization, and storage (CCUS) processes have been widely investigated as promising alternatives to reduce emissions from industries and thermal power plants.^1–3^ Recent reviews demonstrate that absorption, adsorption, gas–solid reaction, cryogenic, and membrane-based methods constitute effective routes to remove CO_2_ from gas streams and even directly from air, achieving efficiencies close to 90% in optimized systems.^3,4^ Nevertheless, technical and economic challenges remain, particularly the high energy requirements associated with the regeneration of conventional sorbents.^5,6^

CCUS technology has evolved from simple geological sequestration strategies to integrated pathways aiming to transform captured CO_2_ into value-added products such as fuels and chemical feedstocks.^2,4,7^ These processes not only mitigate the environmental impact of emissions but also introduce a perspective of a circular carbon economy, converting a waste molecule into a useful raw material.^1,8^ However, traditional approaches—based on amine solvents or solid adsorbents—require high regeneration energy and often suffer from chemical degradation over multiple cycles.^5,6^ Therefore, new solutions combining low regeneration energy, chemical stability, and sustainability have become imperative.

In this context, biomolecules have emerged as innovative alternatives for CO_2_ capture. Peptides and amino acids, for instance, exhibit reactivity toward CO_2_ analogous to that of the natural enzyme RuBisCO, which fixes CO_2_ through carbamate formation.^9^ Recent studies have shown that aqueous solutions of oligopeptides such as diglycine efficiently absorb CO_2_ directly from air, especially when combined with crystalline guanidines that promote regeneration under mild heating (100–120 °C).^9,10^ This hybrid approach, involving bicarbonate crystallization, significantly reduces the regeneration energy compared with conventional solvent-based systems.^9,10^ The specific interaction capacity between amino acid side chains and CO_2_ molecules has also been characterized through spectroscopic and quantum-chemical methods, revealing that polar and charged residues exhibit higher electrostatic affinity for the gas.^11^ These findings pave the way for the rational design of synthetic peptides or modified proteins with enhanced adsorption efficiency and selectivity.^11^ In parallel, the biointegrated CO_2_ capture (BICCU) concept proposes the use of methanogenic microorganisms to convert captured CO_2_ into energy carriers, eliminating the need for energy-intensive desorption stages.^12^ Such biological integration underscores the potential of biomolecule-based platforms for sustainable carbon cycles.

Beyond chemical aspects, recent literature shows that self-organized peptides can form solid materials with high surface area and structural stability suitable for CO_2_ adsorption. Amyloid fibers and peptide nanotubes have been reported as capable of selectively chemisorbing CO_2_ through reversible carbamate formation while maintaining functionality even under humid conditions.^13,14^ These materials can be thermally regenerated with high cycling stability and low energy consumption, representing a notable advancement over traditional amine-based systems.^13,14^ Another relevant advancement is the use of metalized peptide fibrils, which combine metal-coordination sites and enzymatic activities within prion-like self-assembled structures, conferring catalytic properties and chemical selectivity.^15^ This behavior reinforces the versatility of functionalized peptide systems, capable not only of capturing CO_2_ but also of promoting its subsequent conversion. Samples containing divalent cations, for instance, exhibited activities analogous to carbonic anhydrase, suggesting their potential use in hybrid capture–conversion reactors.^15^ From a structural standpoint, amphiphilic and surfactant-like peptides (SLPs) represent a promising class for the development of ordered and self-supported membranes. These systems spontaneously self-assemble through noncovalent interactions—hydrogen bonding, hydrophobic interactions, and electrostatic forces—forming nanotubes, nanoribbons, or stable films in aqueous environments.^12,16–19^ This self-assembling capacity imparts high adaptability and tunability, allowing control over morphology, porosity, and chemical properties in response to pH, ionic strength, and solution composition.^19,20^ The combination of such features with reactive groups capable of carbamate formation positions self-assembled peptide membranes as ideal candidates for selective CO_2_ capture.

Given this background, in contrast to another works, the present study focuses on self-assembled peptide membranes formed by surfactant-like A_6_H and A_6_R sequences whose β-sheet supramolecular structures have been experimentally and theoretically validated.^21–24^ The study aims to correlate the supramolecular structure of peptide films with their CO_2_ adsorption efficiency and surface charge density. The proposal is grounded in recent experimental evidence on the performance of peptides, amyloid fibers, and nanotubes in the controlled fixation and release of CO_2_,^9,13,14^ integrating these insights with molecular self-assembly and bioinspired design principles toward the development of functional peptide-based materials for carbon capture. The choice of A_6_H and A_6_R peptides is motivated by their role as minimal, well-defined model systems for probing the effect of terminal residue chemistry on gas–surface interactions at self-assembled peptide membranes. Both sequences share an identical alanine-rich backbone that promotes β-sheet formation and membrane assembly, while differing exclusively in the chemical nature and charge regulation of the terminal residue. Histidine and arginine were selected because they represent chemically distinct CO_2_-interacting functionalities with markedly different acid–base properties.

## 2 Computational Details

### 2.1 Description of the A_6_H and A_6_R Peptide Membrane System

The A_6_H peptide membrane represents a model amphiphilic nanostructure composed of six alanine (A) residues forming a hydrophobic tail and a single histidine (H) residue providing a hydrophilic headgroup. This asymmetry induces spontaneous self-assembly in aqueous environments, leading to the formation of stable bilayer membranes or nanosheets depending on concentration and pH conditions.^21^ Molecular dynamics (MD) and quantum chemical studies revealed that these membranes are stabilized by a network of hydrogen bonds, Coulombic interactions, and van der Waals forces, which maintain a well-defined hydrophobic core of interdigitated alanine chains, while histidine residues remain exposed to the solvent.^21^ The membranes exhibit thicknesses around 2.3–2.7 nm, consistent with experimental observations of bilayer-like organization, and present small hydrophilic channels through which water molecules can permeate in a controlled manner.^21^ The imidazole ring of histidine, with pKa ≈ 6, enables protonation–deprotonation equilibrium near physiological pH, providing potential sites for CO_2_ binding via carbamate formation and for coordination with transition metal cations, such as Zn^2+^, enhancing their catalytic and adsorption properties.^21^

From a computational perspective, recent MD investigations explored how periodic boundary conditions (PBCs) and system dimensions influence the physicochemical behavior of A_6_H membranes.^25^ These analyses demonstrated that membrane simulations with small surface areas may yield nonconvergent energetic and structural properties, mainly due to artificial constraints at the boundaries. Larger surface models, however, reproduce more realistic molecular mobility and hydrogen-bond dynamics, showing up to a 19% increase in hydrogen-bond lifetime and more accurate peptide tilt and packing when free from PBC artifacts.^25^ The comparison between systems of different sizes also revealed that Coulombic interactions between histidine residues and water molecules intensify as the surface area increases, indicating that larger membranes better capture the cooperative hydration processes that stabilize the peptide–water interface.^25^ These findings are crucial for understanding peptide–gas and peptide–solvent interactions under realistic conditions, such as those expected in CO_2_ adsorption studies.

Given these structural and dynamical characteristics, the A_6_H membrane emerges as an ideal platform for investigating CO_2_ adsorption at the molecular level. Its amphiphilic architecture creates a chemically heterogeneous surface, with hydrophilic histidine-rich regions capable of forming reversible chemical bonds with CO_2_, and hydrophobic alanine domains that stabilize the structural matrix. Furthermore, the well-ordered β-sheet conformation, verified both experimentally and theoretically,^21,25^ provides a regular arrangement of binding sites and controlled diffusion channels for gas molecules. In this context, the present study aims to explore how the surface charge density, pH conditions, and molecular organization of A_6_H peptide membranes modulate CO_2_ adsorption dynamics. By combining molecular dynamics and quantum-mechanical analyses, we seek to elucidate the microscopic mechanisms governing the interaction between CO_2_ and histidine-functionalized membranes, providing fundamental insight for designing bioinspired, peptide-based materials for carbon capture.

In addition to the histidine-based system, the A_6_R peptide membrane^22,23,26^ will also be investigated to assess the effect of the polar headgroup (R) on CO_2_ adsorption. The A_6_R peptide, composed of six alanine (A) residues and a terminal arginine (R), forms similar β-sheet assemblies stabilized by extensive hydrogen-bond networks, but differs markedly in surface charge and electrostatic distribution due to the guanidinium group of arginine.^21^ Previous studies have shown that arginine-rich peptides can generate stronger interfacial electric fields and promote enhanced ion–dipole interactions compared to histidine-containing systems.^21,25^ Evaluating CO_2_ adsorption on both A_6_H and A_6_R membranes will therefore allow a direct comparison between imidazole- and guanidinium-terminated interfaces, providing a deeper understanding of how the nature and protonation state of the terminal residue modulate adsorption strength, orientation, and overall membrane–gas interactions.

The lateral dimensions of the simulation boxes were defined based on a compromise between computational efficiency and the need to minimize finite-size and periodic boundary condition effects. The membrane surface area was chosen to be sufficiently large to ensure proper accommodation of the self-assembled peptide structures without artificial correlation between periodic images, while remaining computationally tractable. Smaller surface areas are known to induce spurious electrostatic and structural artifacts in peptide membrane simulations, whereas excessively large systems dramatically increase computational cost without providing additional physical insight.^21,25^ Consequently, the number of peptide chains (100 for A_6_H and 144 for A_6_R) was determined by the intrinsic packing density and equilibrium lattice parameters of each membrane, ensuring stable β-sheet organization and comparable peptide surface densities in both systems. The box length along the z-direction was independently defined to provide a sufficiently large vacuum region capable of accommodating an adequate number of CO_2_ molecules, allowing gas–surface interactions to occur without interference from periodic images. Therefore, the total box volumes directly reflect the optimized membrane dimensions and the required free volume for CO_2_ adsorption, rather than an arbitrary choice of system size.

### 2.2 Computational Modeling

Molecular dynamics (MD) simulations were performed using the GROMACS software package,^27,28^ widely employed for atomistic modeling of biomolecular and self-assembled materials. Simulation boxes were built with Packmol,^29^ ensuring a random and homogeneous distribution of CO_2_ molecules around the A_6_H and A_6_R peptide membrane. The initial system consisted of a pre-equilibrated A_6_H or A_6_R peptide membrane positioned at the center of the box and fully solvated in water or water and Cl^–^ ions. After the thermodynamic equilibration process, a long production run was conducted to obtain a structurally stable A_6_H and A_6_R membranes. Subsequently, water molecules were removed and replaced by CO_2_ molecules to evaluate CO_2_ adsorption by the A_6_H and A_6_R membranes under idealized (vacuum condition) conditions.

It is important to clarify that the CO_2_ adsorption stage was intentionally modeled under idealized conditions, in which water molecules were removed after membrane equilibration and CO_2_ molecules were introduced in a vacuum region. This approach isolates the intrinsic interaction mechanisms between CO_2_ and the peptide membrane, allowing a direct assessment of the role of terminal residue chemistry, electrostatics, and supramolecular organization without the competing effects of solvent or other gas species. In real conditions, such as atmospheric environments (CO_2_ diluted in N_2_/O_2_ mixtures) or aqueous media, additional factors, including competitive adsorption, solvent screening, and chemical equilibria (e.g., CO_2_ hydration to carbonic acid), would influence the adsorption behavior. These effects are expected to reduce the effective interaction strength and modify the spatial distribution of CO_2_ at the interface. Therefore, the present simulations should be interpreted as a fundamental, mechanistic study that establishes the upper-limit interaction regimes and intrinsic adsorption characteristics of the peptide membranes. This simplified framework provides a controlled reference for understanding how peptide chemistry governs CO_2_ affinity, which is a necessary step before introducing more complex and realistic environmental conditions in future investigations.

All molecular dynamics simulations were performed using the CHARMM36 all-atom force field, which has been extensively validated for peptides, proteins, and charged amino acid side chains in aqueous and interfacial environments.^30–33^ The A_6_H and A_6_R peptide membranes were modeled using standard CHARMM36 topology and parameter files, with histidine and arginine described in their dominant protonation states at physiological pH. Water molecules were represented using the TIP3P model^34,35^ and were included exclusively during the membrane self-assembly and equilibration stages. Carbon dioxide molecules were modeled using a standard CHARMM-compatible three-site representation with fixed partial charges and Lennard-Jones interactions. This type of model is widely employed in MD simulations and provides an adequate description of the quadrupolar character of CO_2_, which is the dominant factor governing its intermolecular and interfacial interactions. In the context of the present work, this representation is appropriate for capturing relative trends in CO_2_–peptide interactions and for comparing the adsorption behavior between different membrane chemistries under consistent simulation conditions. Chloride ions were described using CHARMM36 ion parameters consistent with the TIP3P water model. A summary of the system composition and particle numbers is provided in Table 1. Table 1 lists the total number of particles used in each simulation. Long-range electrostatic interactions were calculated using the Particle Mesh Ewald (PME) method^36^ with a cutoff radius of 1.2 nm, whereas van der Waals forces were computed using the cutoff method with a potential-shift-Verlet modifier. Covalent bonds were constrained using the LINCS algorithm,^37^ allowing an integration time step of 1.0 fs (0.001 ps). The target temperature of 300 K was maintained using the v-rescale thermostat^38^ with a coupling constant of 0.2 ps, while the pressure was controlled at 1.013 bar by a semi-isotropic Parrinello–Rahman barostat,^39^ using a compressibility of 4.5 × 10^–5^ bar^–1^ and a coupling constant of 2 ps—conditions essential for preserving membrane integrity. During the membrane–CO_2_ simulations, the simulation box volume was kept constant (NVT ensemble), allowing CO_2_ molecules, initially distributed randomly, to spontaneously interact with the peptide surface under vacuum conditions. All simulations were conducted under three-dimensional periodic boundary conditions (PBCs) to reproduce the behavior of an extended surface. The initial peptide structure was extracted from the β-sheet conformation experimentally described for A_6_H, characterized by parallel strand alignment and antiparallel stacking between layers, as previously modeled in ref 25.

**Table 1:** Simulation Parameters for the A_6_H and A_6_R Peptide Membranes Used in CO_2_ Adsorption Studies^a^

Membrane Type	# Peptide	Box Area (nm^2^)	Box Volume (nm^3^)	Pep/Area	# CO_2_	# Cl^–^	σ_S_ (e/nm^2^)
A_6_H	100	26.84	242.92	3.73	39	–	0.0000
A_6_R	144	41.89	350.33	3.44	50	144	1.7188

a The table reports the number of peptides, simulation box dimensions, peptide surface density, and number of CO_2_ and Cl^–^ molecules included in each system. The surface charge density (σ_S_) reflects the charge contribution from the terminal amino acid residue (histidine or arginine) and defines the electrostatic environment relevant to CO_2_ adsorption. Surface charge densities were computed after equilibration as the net charge of surface-exposed terminal residues divided by the membrane surface area.

Protonation states of the terminal residues (H and R) were assigned based on their dominant forms under near-neutral conditions. Arginine residues were modeled in their fully protonated guanidinium form, consistent with their high pKa. Histidine residues were modeled in their neutral imidazole form, which represents the predominant state at pH 7, while recognizing that partial protonation may occur depending on the local environment. As the primary objective of this work is a comparative assessment of CO_2_ adsorption at ordered peptide membranes, fixed protonation states were adopted to ensure consistency and to isolate the effect of terminal residue chemistry and supramolecular organization. Charge neutrality was ensured based on the intrinsic chemical nature and protonation state of the terminal residues in each membrane. In the A_6_R system, the terminal arginine residues adopt their naturally protonated guanidinium form under near-neutral conditions, resulting in a net positive charge of the membrane. To maintain electrostatic neutrality of the simulation box, chloride counterions were therefore added. In contrast, in the A_6_H membrane the histidine residues were modeled in a protonated imidazole form that carries no net charge, leading to an overall neutral membrane. Consequently, no counterions were required for the A_6_H system.

The membrane construction and stabilization protocol consisted of two main stages: (a) thermodynamic equilibration and (b) production. In the equilibration stage, a series of short simulations (∼5 ns each) were performed, alternating between the NVT and NPT ensembles until the system achieved stability in potential energy, density, and surface area (totaling approximately 50 ns). The production phase then consisted of a continuous 100 ns NPT simulation, recording 50,000 configurations for the statistical analysis. This methodology accurately reproduces the theoretical and experimental behavior of the A_6_H and A_6_R membrane in aqueous/aqueous-ionic environments,^25^ forming a solid basis for the subsequent CO_2_ adsorption stage, in which water molecules were removed and replaced by CO_2_. This final phase was performed in the NVT ensemble for 200 ns, divided into segments of 100 ns each (saving 50% of configurations), under identical simulation conditions. Finally, all structural and dynamic analyses were carried out using GROMACS tools.^27,28^ Visual inspection and three-dimensional rendering of the simulated systems were performed with VMD.^40^ This computational setup ensures statistical consistency and structural reliability for investigating the molecular mechanisms governing CO_2_ adsorption on the self-assembled A_6_H and A_6_R peptides membranes, providing an atomistic representation of the adsorption process. Figure 1 shows A_6_H and A_6_R peptide membranes, CO_2_, Cl^–^ ions in initial structures.

**Figure 1:**
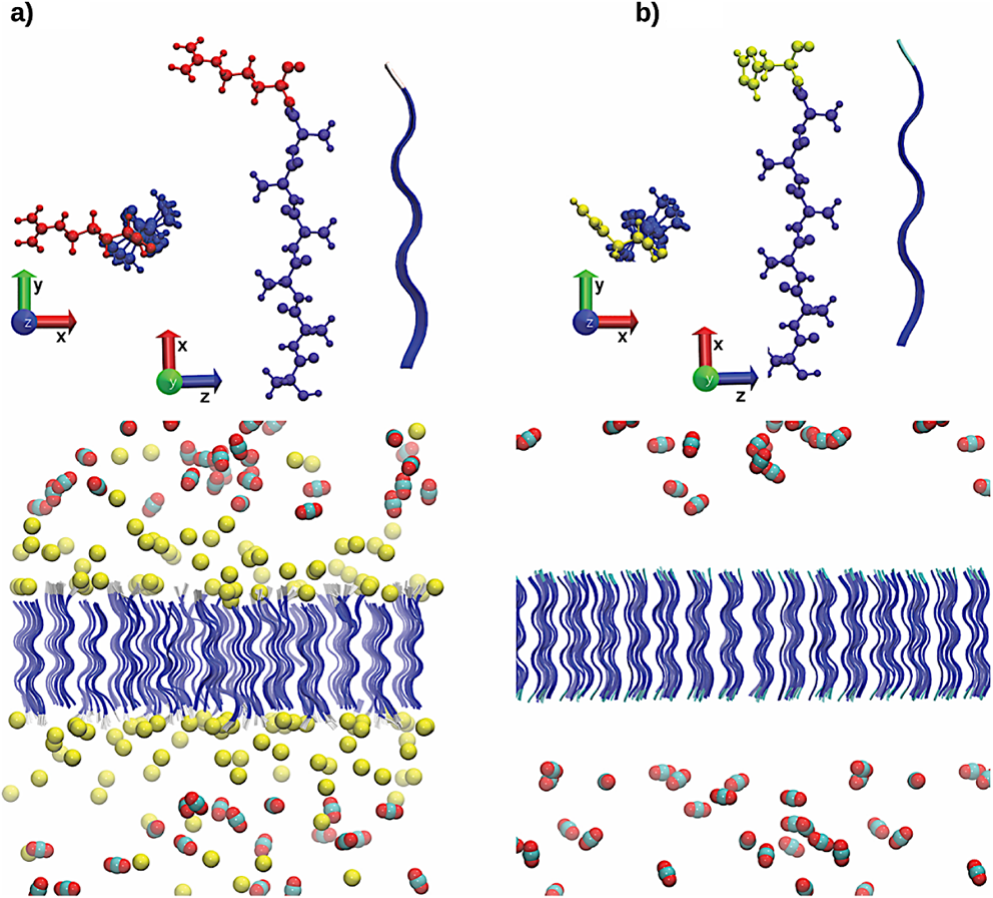
Initial configurations of the peptide nanomembrane systems: (a) A_6_R and (b) A_6_H. The upper panels show representative molecular structures of the peptide chains, highlighting the terminal residues (arginine in A_6_R and histidine in A_6_H). The lower panels present the full simulation boxes, including the self-assembled peptide membranes, CO_2_ molecules, and, in the case of A_6_R, chloride counterions. The membranes exhibit well-organized β-sheet structures with the hydrophobic alanine core and hydrophilic terminal residues exposed at the interface. Color code: peptide backbone = blue ribbons; terminal residues = atomistic representation; CO_2_ molecules = red (oxygen) and gray (carbon); chloride ions = yellow. The coordinate axes are shown for reference.

## 3 Results and Discussion

The Results and Discussion section is organized to establish a clear mechanistic connection between membrane structure, interfacial chemistry, and CO_2_ adsorption behavior. We first characterize the structural organization and surface charge properties of the A_6_H and A_6_R membranes, then examine peptide–CO_2_ interaction energies and interfacial density distributions, and finally analyze hydrogen bonding dynamics and CO_2_ mobility to provide an integrated molecular-level interpretation of adsorption mechanisms.

### 3.1 Energy Interaction

The analysis of the average interaction energies (Table 2) reveals a scenario in which the predominant forces in the system are clearly those associated with peptide–peptide interactions (A_6_H–A_6_H and A_6_R–A_6_R). In both membranes, these contributions are 2 orders of magnitude greater than the peptide–CO_2_ interactions and three orders above the CO_2_–CO_2_ interactions, highlighting the dominant role of intramembrane cohesion in structural stabilization. In absolute terms, the electrostatic component represents the largest fraction of the stabilization energy for both A_6_H and A_6_R structures, with strongly negative values, whereas the Lennard-Jones terms contribute complementarily, reflecting favorable dispersive contacts among compact side chains, as expected. In contrast, the CO_2_–CO_2_ and CO_2_–ion (when present) interactions are energetically modest, confirming that gas adsorption is highly dependent on the peptide surface sites rather than on spontaneous CO_2_ self-aggregation at the interface. Although small compared to the intrinsic forces of the membrane, the peptide–CO_2_ interactions remain consistently attractive in both peptides, indicating a spontaneous affinity of CO_2_ for the membrane surface. For A_6_H, the average total interaction energy is approximately −11.2 kJ·mol^–1^ per peptide, obtained as the sum of the Coulomb (−6.2 kJ·mol^–1^) and Lennard-Jones (−5.1 kJ·mol^–1^) contributions. Similarly, for A_6_R, the total interaction energy is ≈− 8.5 kJ·mol^–1^ per peptide, resulting from the sum of Coulomb (−3.0 kJ·mol^–1^) and Lennard-Jones (−5.5 kJ·mol^–1^) terms. The CO_2_–CO_2_ contributions, ranging between −0.3 and −0.3 kJ·mol^–1^ per CO_2_, corroborate that intermolecular aggregation of the gas is negligible compared to interactions with the peptide surface, reinforcing the character of dispersed, localized adsorption at the supramolecular interface.

**Table 2:** Coulomb and Lennard-Jones Interaction Energies for the A_6_H and A_6_R Peptide Membranes Interacting with CO_2_ and, for A_6_R, Chloride Ions^a^

	0–100 ns		100–200 ns		Average	
System	Coulomb	Lennard-Jones	Coulomb	Lennard-Jones	Coulomb	Lennard-Jones
A_6_H–A_6_H	–2760.7 ± 2.5	–174.5 ± 1.4	–2761.5 ± 2.6	–174.1 ± 1.4	–2761.1 ± 2.6	–174.3 ± 1.4
A_6_H–CO_2_	–6.2 ± 0.4	–5.2 ± 0.3	–6.1 ± 0.4	–5.1 ± 0.3	–6.2 ± 0.4	–5.1 ± 0.3
CO_2_–CO_2_	–0.3 ± 0.1	–0.3 ± 0.1	–0.3 ± 0.2	–0.3 ± 0.1	–0.3 ± 0.1	–0.3 ± 0.1

System	Coulomb	Lennard-Jones	Coulomb	Lennard-Jones	Coulomb	Lennard-Jones
A_6_R–A_6_R	–1941.2 ± 2.7	–154.3 ± 1.2	–1941.2 ± 2.7	–154.9 ± 1.2	–1941.2 ± 2.7	–154.6 ± 1.2
A_6_R–CO_2_	–3.0 ± 0.3	–5.6 ± 0.3	–3.00 ± 0.3	–5.5 ± 0.3	–3.0 ± 0.3	–5.5 ± 0.3
A_6_R–Ions	–353.0 ± 2.0	32.8 ± 1.0	–353.7 ± 1.9	33.0 ± 1.0	–353.4 ± 1.9	32.9 ± 1.0
CO_2_–CO_2_	–0.4 ± 0.1	–0.3 ± 0.1	–0.4 ± 0.1	–0.3 ± 0.1	–0.4 ± 0.1	–0.3 ± 0.1
CO_2_ *–*Ions	–1.2 ± 0.6	–0.7 ± 0.2	–1.3 ± 0.6	–0.7 ± 0.2	–1.3 ± 0.6	–0.7 ± 0.2

a Values correspond to two sequential 100 ns simulation windows (0–100 ns and 100–200 ns) and the averaged contributions. Interaction components include peptide–peptide, peptide–ion, and peptide–CO_2_ (kJ·mol^–1^ per peptide); CO_2_–CO_2_ and CO_2_–ion interactions (kJ·mol^–1^ per CO_2_). The results highlight the differences in electrostatic and van der Waals interactions driven by the peptide terminal residue (histidine vs. arginine), revealing stronger peptide–peptide stabilization in A_6_H systems and enhanced ion-mediated electrostatic contributions in A_6_R systems. Error values denote standard deviations. Interaction energies represent average peptide–CO_2_ affinities per peptide and are not directly comparable to macroscopic adsorption capacities. Values are reported as mean ± standard deviation.

The comparison between the 0–100 ns and 100–200 ns simulation intervals demonstrates high temporal stability across all energetic channels. Average fluctuations remain within the standard deviations, and no drift is observed, indicating that the systems reached a stationary regime suitable for thermodynamic and structural analysis. This robust convergence implies that the membranes remained structurally equilibrated throughout the simulations and that the presence of CO_2_ did not induce global perturbations capable of altering the regime of intermolecular interactions. Consequently, the mean values faithfully represent the statistical state of the system and can be interpreted as descriptors of a dynamic equilibrium adsorption state. A breakdown by interaction type reinforces this global stability: peptide–peptide interactions in both systems vary by less than 1 kJ·mol^–1^ (per peptide) in their electrostatic components—negligible relative to their magnitude—while peptide–CO_2_ interactions fluctuate by less than 0.1 kJ·mol^–1^ per peptide, confirming that the adsorption process remains dynamically equilibrated without significant reorganization over time. Likewise, CO_2_–CO_2_ and CO_2_–ion interactions exhibit stability consistent with statistical noise, suggesting the absence of relevant cooperative processes or ion rearrangement induced by the gas.

Comparing the two membranes reveals that the A_6_H structure exhibits a more negative intrapeptide cohesion energy (−2761 kJ·mol^–1^ per peptide) than A_6_R (−1941 kJ·mol^–1^ per peptide) for both electrostatic and dispersive contributions. This difference indicates that A_6_H forms a more strongly stabilized structure, implying a stiffer interface that is less dependent on external compensation. In contrast, the A_6_R system shows significant interaction energy with chloride ions (≈−353 kJ·mol^–1^ per peptide), indicating that its electrostatic stability is partly mediated by counterions, as expected for highly charged surfaces bearing guanidinium groups. This finding suggests a crucial role of ionic species in the interfacial behavior of A_6_R, with potential implications for CO_2_ access to the active sites. When comparing peptide–CO_2_ affinity directly, A_6_H shows a larger electrostatic contribution than A_6_R (−6.2 vs −3.0 kJ·mol^–1^ per peptide), while the Lennard-Jones term is slightly more attractive for A_6_R (−5.5 vs −5.1 kJ·mol^–1^ per peptide). These results indicate that histidine favors the electrostatic recognition of CO_2_, likely due to its polarizability and directional stabilization capacity, whereas arginine promotes more dispersive but less selective interactions, owing to electrostatic competition with Cl^–^. Therefore, the chemistry of the terminal residue emerges as a key determinant of adsorption selectivity and strength. The strong A_6_R–Cl^–^ electrostatic attraction, accompanied by a positive Lennard-Jones repulsion, demonstrates that the gas does not predominantly compete with ions for the interface but rather that the ions spontaneously occupy the most favorable electrostatic sites, reducing the local potential available for CO_2_. This electrostatic screening explains the lower magnitude of peptide–CO_2_ interactions in the A_6_R system and highlights the importance of the ionic microenvironment in modulating gas affinity. Overall, our results show that the A_6_H membrane possesses a more cohesive structure and an electrostatically favorable interface for spontaneous CO_2_ adsorption, whereas the A_6_R membrane, although still capable of gas interaction, exhibits significant electrostatic competition due to strong counterion association. Both surfaces adsorb CO_2_ stably and consistently over 200 ns but through distinct mechanisms: A_6_H is driven by distributed electrostatics and local charge density, while A_6_R is modulated by ionic screening and dispersive effects, underscoring that the terminal-group chemistry of peptides is a structural parameter in designing bioinspired membranes for selective CO_2_ capture.

### 3.2 Mass Density Profile

To translate these interaction energies into spatial adsorption behavior, we analyze the interfacial distribution and accumulation of CO_2_ molecules near the membrane surfaces using number density profiles. Figure 2 shows the mass density profiles (kg·m^–3^) along the z-axis for the A_6_H and A_6_R peptide membranes interacting with CO_2_ and, for the A_6_R system, with chloride ions. Panels (a–d) correspond respectively to the 0–100 ns and 100–200 ns simulation intervals for both peptide types. In all cases, the density distributions are centered along the membrane midplane, where the sharp peaks correspond to the peptide-rich regions defining the hydrophobic bilayer core. The shaded yellow regions represent the membrane structure and thickness determined from the distance between the density profile of opposite leaflets for 600 kg·m^–3^. The mass profiles of CO_2_ and ions are superimposed to reveal their spatial localization relative to the membrane surface, allowing an assessment of adsorption, penetration, and temporal stability during the two simulation windows.

**Figure 2:**
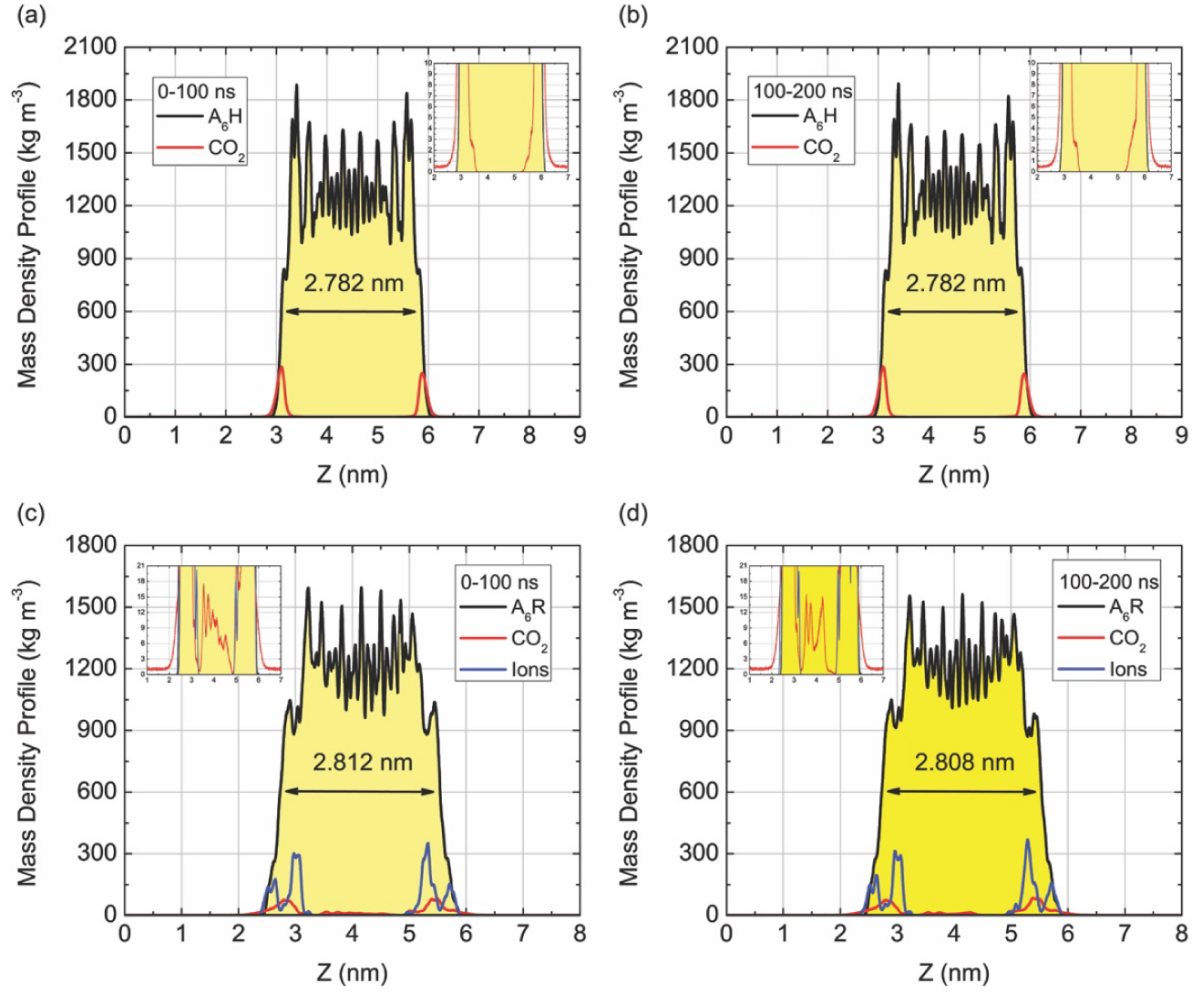
Mass density profiles (kg·m^–3^) along the z-axis for the A_6_H and A_6_R peptide nanomembranes interacting with CO_2_ and, in the case of A_6_R, with chloride ions. Panels (a) and (b) correspond to the A_6_H–CO_2_ system, while (c) and (d) represent the A_6_R–CO_2_–Cl^–^ system, each evaluated over two consecutive 100 ns simulation intervals (0–100 ns and 100–200 ns). The black curves represent the peptide density, the red curves correspond to CO_2_, and the blue curves (for A_6_R) indicate the distribution of chloride ions. The shaded yellow region denotes the nanomembrane mass, whose average thickness is indicated within each panel (≈2.78 nm for A_6_H and ≈2.81 nm for A_6_R). Insets show magnified views of the CO_2_ density near the membrane interface. Both peptide nanomembranes maintain structural stability and thickness over 200 ns, while CO_2_ molecules preferentially localize at the outer interfaces, exhibiting surface adsorption behavior.

For the A_6_H membrane (Figure 2a–b), the density profiles of the peptide show a symmetric and well-defined bilayer structure with an average thickness of approximately 2.782 nm, which remains unchanged between the two simulation intervals. The CO_2_ density curve exhibits distinct peaks at the membrane–vacuum interface, indicating preferential surface adsorption rather than penetration into the hydrophobic core. The absence of significant changes between 0–100 ns and 100–200 ns demonstrates that the adsorption of CO_2_ molecules reaches equilibrium early in the simulation and remains stable thereafter. These results confirm that the A_6_H structure maintains its supramolecular organization and acts as a robust substrate for sustained CO_2_ adsorption without structural deformation or drift in thickness. In the case of the A_6_R membrane (Figure 2c–d), the peptide density profile also shows a stable and symmetric bilayer organization, with a slightly higher average thickness of ≈2.81 nm. This increase is consistent with the larger steric volume and higher hydration of the guanidinium headgroups at the membrane surface. The CO_2_ distribution is similar to that observed for A_6_H, with adsorption mainly at the outer interfaces. However, a key difference is the presence of chloride ions, whose density peaks overlap partially with the peptide surface. The Cl^–^ ions form a tightly bound layer that partially screens the positive charges of the arginine residues, thereby modulating the local electrostatic potential available for CO_2_ interaction. The resulting profiles reveal that CO_2_ molecules are displaced slightly away from the interface relative to A_6_H, indicating weaker electrostatic stabilization and competition with surface-bound ions.

The comparison between A_6_H and A_6_R membranes highlights distinct physicochemical environments governing gas adsorption. While both systems exhibit structural stability and symmetric density distributions, A_6_H presents a more compact and cohesive nanomembrane, favoring stronger peptide–CO_2_ electrostatic interactions. In contrast, A_6_R displays an interfacial region dominated by counterions, producing an ions-mediated screening effect that weakens direct Coulombic attraction with CO_2_. This interpretation agrees with the energy decomposition analysis, which showed larger Coulombic contributions for A_6_H and a compensating role of dispersive interactions for A_6_R. The mass density results thus reinforce the notion that terminal residue chemistry—imidazole vs guanidinium—controls both the magnitude and spatial localization of CO_2_ adsorption. Together, these observations elucidate how variations in peptide headgroup chemistry can be exploited to design tunable, bioinspired materials for selective carbon capture applications. Figure 2 also demonstrates that both peptide membranes retain their structural integrity and thickness throughout 200 ns of simulation, indicating that CO_2_ adsorption does not perturb the nanomembrane morphology and the constant mass density profiles across time confirm a dynamic equilibrium regime for gas–surface interactions. Figure 3 shows a final molecular dynamics configuration highlighting the structural features observed in the mass density profile.

**Figure 3:**
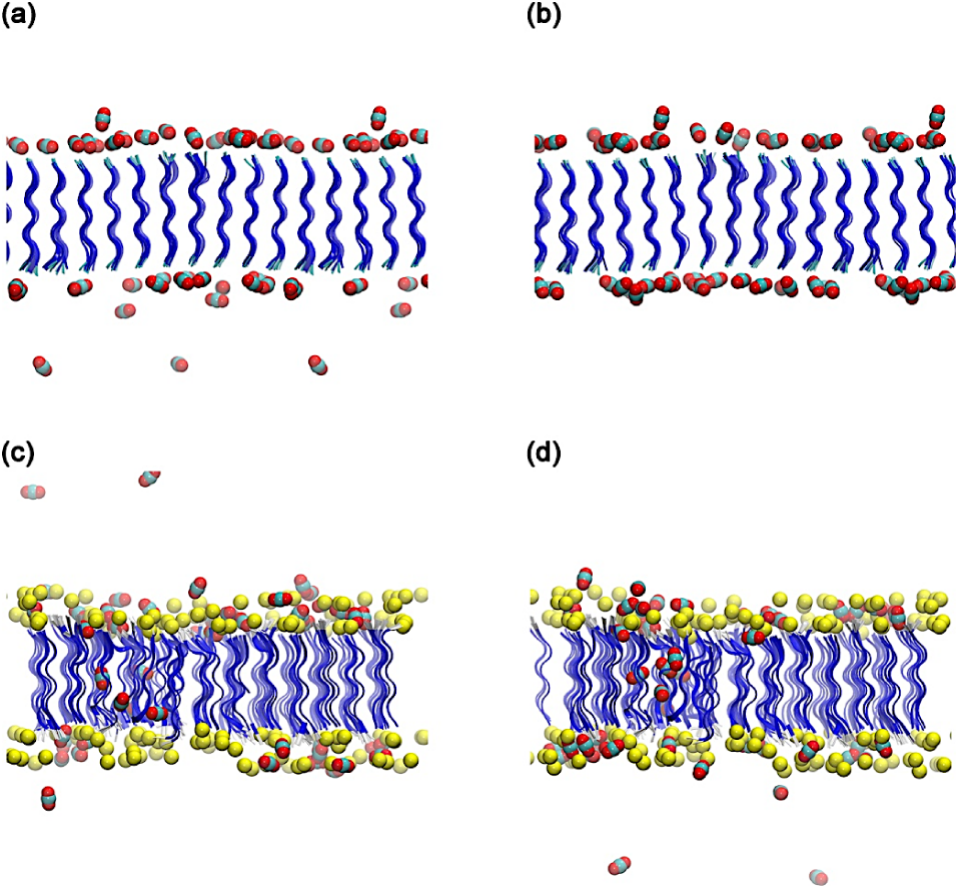
Final MD-configurations of the A_6_H and A_6_R peptide nanomembranes interacting with CO_2_ and, for A_6_R, with chloride ions. Panels (a) and (b) correspond to the A_6_H–CO_2_ system at 100 and 200 ns, respectively, while panels (c) and (d) show the A_6_R–CO_2_–Cl^–^ system for the same time intervals. Blue ribbons represent peptide backbones, red and gray spheres denote CO_2_ molecules (oxygen and carbon, respectively), and yellow spheres indicate chloride ions. The snapshots illustrate the interfacial adsorption of CO_2_ molecules on both peptide surfaces.

### 3.3 Radial Distribution Function and Local Coordination Analysis

To further characterize the local organization of CO_2_ molecules at the peptide interfaces, radial distribution functions (RDFs) were calculated between CO_2_ molecules and the center of mass of the peptides A_6_H or A_6_R. This analysis provides a molecular-level description of preferential distances and local accumulation of CO_2_ at the membrane, complementing the energetic and mass density results discussed previously. The RDF profiles (Figure 4) reveal clear differences in short-range organization between the two systems. In the A_6_R membrane, a nonzero probability of finding CO_2_ molecules is observed at distances below 0.5 nm, whereas for A_6_H this region is essentially inaccessible. This indicates that CO_2_ molecules can approach significantly closer to the guanidinium groups (A_6_R) than to the imidazole rings (A_6_H), consistent with the stronger electrostatic attraction mediated by the positively charged arginine residues and their associated ionic environment.

**Figure 4:**
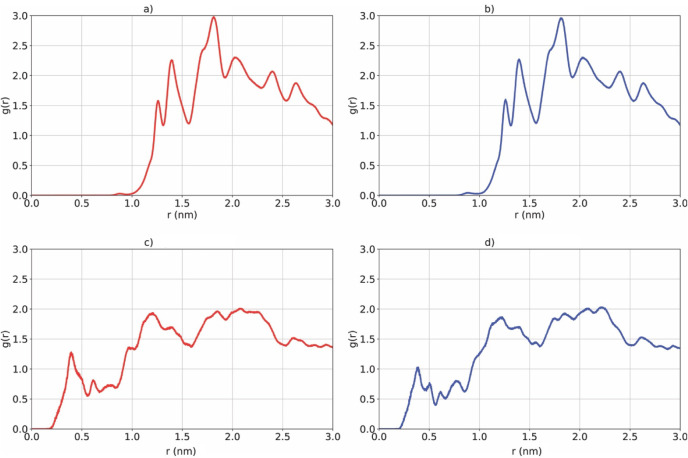
Radial distribution functions (RDFs) between CO_2_ molecules and center of mass of the peptide in nanomembranes: (a) A_6_H–CO_2_ (0–100 ns), (b) A_6_H–CO_2_ (100–200 ns), (c) A_6_R–CO_2_ (0–100 ns), and (d) A_6_R–CO_2_ (100–200 ns).

Integration of the RDF curves was performed in a shell-resolved manner, yielding the number of CO_2_ molecules within specific radial intervals relative to the membrane center of mass. Based on this reference, two physically meaningful regions were defined: an inner region (0–1.5 nm), encompassing the membrane core and extending up to its surface, and an outer region (1.5–2.5 nm), corresponding to the interfacial adsorption layer beyond the membrane surface. In the inner region (0–1.5 nm), the A_6_R system exhibits a significantly higher CO_2_ population compared to A_6_H. For A_6_R, the total number of CO_2_ molecules in this region is approximately 1.78 (0–100 ns) and 1.67 (100–200 ns), whereas for A_6_H the values are markedly lower, around 0.56 (0–100 ns) and 0.56 (100–200 ns). This indicates that CO_2_ molecules penetrate closer to the membrane interior in A_6_R, reflecting a stronger attraction toward the charged guanidinium groups and the associated electrostatic environment.

In contrast, in the outer region (1.5–2.5 nm), both systems display comparable CO_2_ populations, although A_6_H shows a slightly higher accumulation. The A_6_R system presents approximately 16.71 (0–100 ns) and 16.49 (100–200 ns) molecules, while A_6_H reaches approximately 18.31 (0–100 ns) and 18.34 (100–200 ns). This suggests that, beyond the membrane surface, CO_2_ distribution becomes more homogeneous, with a tendency for slightly greater accumulation in the A_6_H system due to its more diffuse and less restrictive interfacial environment. The comparison between the two simulation intervals confirms that these distributions are highly stable over time, indicating a well-converged structural organization. Overall, the RDF analysis reveals that the primary distinction between the systems lies in the inner region: A_6_R promotes a deeper and more structured penetration of CO_2_ toward the membrane surface, whereas A_6_H favors a more external and diffuse adsorption profile. This provides direct structural evidence that terminal residue chemistry controls the depth of CO_2_ interfacial localization rather than the total extent of adsorption.

Additionally, the RDF-based analysis allows a geometric interpretation of the adsorption regime in terms of the effective distance between CO_2_ molecules and the membrane surface. By defining the membrane surface at approximately 1.5 nm from the center of mass, three distinct spatial regimes can be identified: (i) an inner region (0–1.5 nm), corresponding to surface adsorption and direct interaction with the membrane; (ii) an interfacial region (1.5–2.5 nm), associated with persistent but less localized interactions; and (iii) an outer region (>2.5 nm), where CO_2_ molecules behave as quasi-free species with minimal influence from the membrane. Within this framework, the A_6_R system exhibits a higher population of CO_2_ molecules in the inner region, indicating a greater tendency for direct surface adsorption and stronger interfacial confinement. In contrast, the A_6_H system shows a reduced population in this region and a relative enrichment in the interfacial layer, consistent with a more diffuse adsorption regime dominated by weaker and transient interactions. Therefore, although both systems display stable adsorption behavior, A_6_R is characterized by a more localized and surface-bound regime, whereas A_6_H favors a broader distribution of CO_2_ in the near-interface region, approaching a transition toward free diffusion. This distance-based classification provides a clear geometric description of the adsorption process and complements the energetic and structural analyses discussed above.

### 3.4 Hydrogen Bonding Statistics and Dynamics Analyses

Hydrogen bonds (HBs) between CO_2_ molecules and the A_6_H or A_6_R peptide membranes were identified along the molecular dynamics trajectories using conventional geometric criteria based on distance and angle. A bond was considered an HB when the donor–acceptor distance satisfied d_D–A_ ≤ 0.35 nm and the donor–hydrogen–acceptor angle met the condition θ_D–H···A_ ≤ 40°. These thresholds are well established for biomolecular and supramolecular systems, ensuring that only interactions of genuine stabilizing character are detected while excluding random steric contacts. The analysis was performed over 200 ns of simulation, divided into two 100 ns intervals, allowing the estimation of the average number of HBs per CO_2_ molecule, their mean lifetime (τ), and the Gibbs free energy of bond rupture (ΔG), using Luzar, Chandler and Van Der Spoel theory,^41–43^ which reflects the average strength of the interaction. The Gibbs free energy of bond rupture (ΔG) was estimated based on hydrogen-bond kinetics using the Luzar–Chandler formalism, representing an effective free energy associated with bond stability rather than a standard thermodynamic quantity.

The results in Table 3 show that both systems exhibit nearly identical mean HB frequencies (approximately 0.32 HBs per CO_2_ molecule) indicating that CO_2_ interacts with polar sites on both peptide surfaces with comparable probability. However, this quantitative similarity conceals pronounced qualitative differences in kinetic stability and thermodynamic strength. In A_6_H, the HBs are short-lived and highly dynamic, whereas in A_6_R they are long-lived and more energetically persistent, reflecting two distinct adsorption regimes dominated respectively by transient polarization and by strong electrostatics. Therefore, while the number of interactions remains similar, the nature and temporal stability of those hydrogen bonds diverge significantly between the two peptides.

**Table 3:** Hydrogen-Bond Statistics between CO_2_ Molecules and the A_6_H or A_6_R Peptide Nanomembranes Obtained from 200 ns Molecular Dynamics Simulations^a^

	0–100 ns			100–200 ns		
System	# HBs/# CO_2_	HBs-Lifetime	ΔG	# HBs/# CO_2_	HBs-Lifetime	ΔG
A_6_H–CO_2_	0.32	9.7 ps	10.2 kJ·mol^–1^	0.32	9.7 ps	10.2 kJ·mol^–1^
A_6_R–CO_2_	0.32	341.2 ps	18.9 kJ·mol^–1^	0.33	167.6 ps	17.2 kJ·mol^–1^

a Values correspond to two consecutive 100 ns intervals and include the average number of hydrogen bonds per CO_2_ molecule (#HBs/#CO_2_), the mean HB lifetime (τ), and the Gibbs free energy of bond rupture (ΔG). Hydrogen bonds were defined using a donor–acceptor distance ≤ 0.35 nm and a donor–hydrogen–acceptor angle ≤ 40°.

In the A_6_H–CO_2_ system, HBs display a mean lifetime of 9.7 ps and a ΔG of 10.2 kJ·mol^–1^, values that remain unchanged across both simulation windows. This invariance indicates a transient interaction regime, in which CO_2_ molecules associate briefly with the polar regions of the imidazole ring but rapidly dissociate due to the weak anchoring strength. The constancy of the parameters suggests that the system attains dynamic equilibrium early in the trajectory, with no major structural rearrangements. Such short-lived HBs are consistent with the mass-density profiles (Figure 2), which showed stable yet superficial adsorption. The A_6_H membrane thus provides a flexible and adaptive interfacial environment, promoting reversible CO_2_ binding without compromising molecular mobility or membrane integrity. In contrast, the A_6_R–CO_2_ system exhibits markedly longer HB lifetimes, reaching 341 ps in the first 100 ns and decreasing to 167 ps in the second interval, with corresponding ΔG values of 18.9 and 17.2 kJ·mol^–1^. These data indicate stronger, cooperative hydrogen bonds, likely formed between the positively charged guanidinium groups and the oxygen atoms of CO_2_, partially reinforced by local electrostatic fields generated by chloride counterions. The stronger and longer-lived HBs in A_6_R model are consistent with its more negative electrostatic interaction energy (≈−353 kJ·mol^–2^ per peptide including ion effects), confirming that ion-mediated fields not only stabilize peptide–peptide interactions but also enhance peptide–CO_2_ coupling. The reduction in lifetime during the later stage reflects interfacial reorganization, where initial strong associations give way to a balance of shorter, less stable interactions as the ion distribution and CO_2_ population reach equilibrium. The system thus evolves toward a steady-state adsorption regime governed by ion-mediated electrostatics.

The ≈8 kJ·mol^–1^ difference in ΔG between the two systems demonstrates that the HBs formed at the A_6_R interface are thermally more resistant to disruption. This behavior results from the electrostatic screening produced by chloride ions, which partially neutralize the guanidinium charges and generate local electric fields that intensify interactions with CO_2_. In A_6_H, by contrast, the neutral imidazole groups favor transient dipole-induced interactions that are weaker but more dynamically accessible. Consequently, A_6_H behaves as a dynamic, regenerable adsorbent, while A_6_R acts as a rigid, charge-regulated surface with higher binding energy but slower desorption kinetics. These complementary behaviors emphasize that peptide surface charge governs both the strength and reversibility of gas adsorption. The hydrogen-bond analysis underscores that CO_2_ adsorption at peptide membranes is not solely determined by the number of polar sites, but by the electrostatic topology and cooperative ion effects that dictate the persistence of intermolecular interactions. Such findings highlight the potential of peptide membranes as tunable molecular platforms, where controlled variation in charge distribution and side-chain polarity can be strategically employed to optimize gas capture efficiency, selectivity, and reversibility in bioinspired carbon-sequestration materials.

Figure 5 presents the hydrogen-bond (HB) network established between CO_2_ and peptides molecules and between peptides in nanomembranes A_6_H and A_6_R after 200 ns of molecular dynamics simulation, shown from two orthogonal perspectives: the zx (left) and zy (right) planes. In the A_6_H–CO_2_ system, the red dashed lines illustrate spatially localized HBs concentrated at the outer surfaces of the membrane, where the imidazole residues of histidine interact intermittently with the CO_2_ oxygens. These bonds form a discontinuous and flexible network, consistent with the short HB lifetimes and low ΔG values reported in Table 3. Conversely, in the A_6_R–CO_2_–Cl^–^ system, a denser and more interconnected HB pattern emerges, extending along both planar orientations. The presence of chloride ions induces electrostatic stabilization and cooperative alignment of the guanidinium groups, which results in stronger and longer-lived HBs with CO_2_. The comparison between the two peptides highlights how interfacial charge distribution controls both the geometry and persistence of the hydrogen-bond network, confirming that the A_6_R surface provides a more electrostatically structured environment for CO_2_ retention.

**Figure 5:**
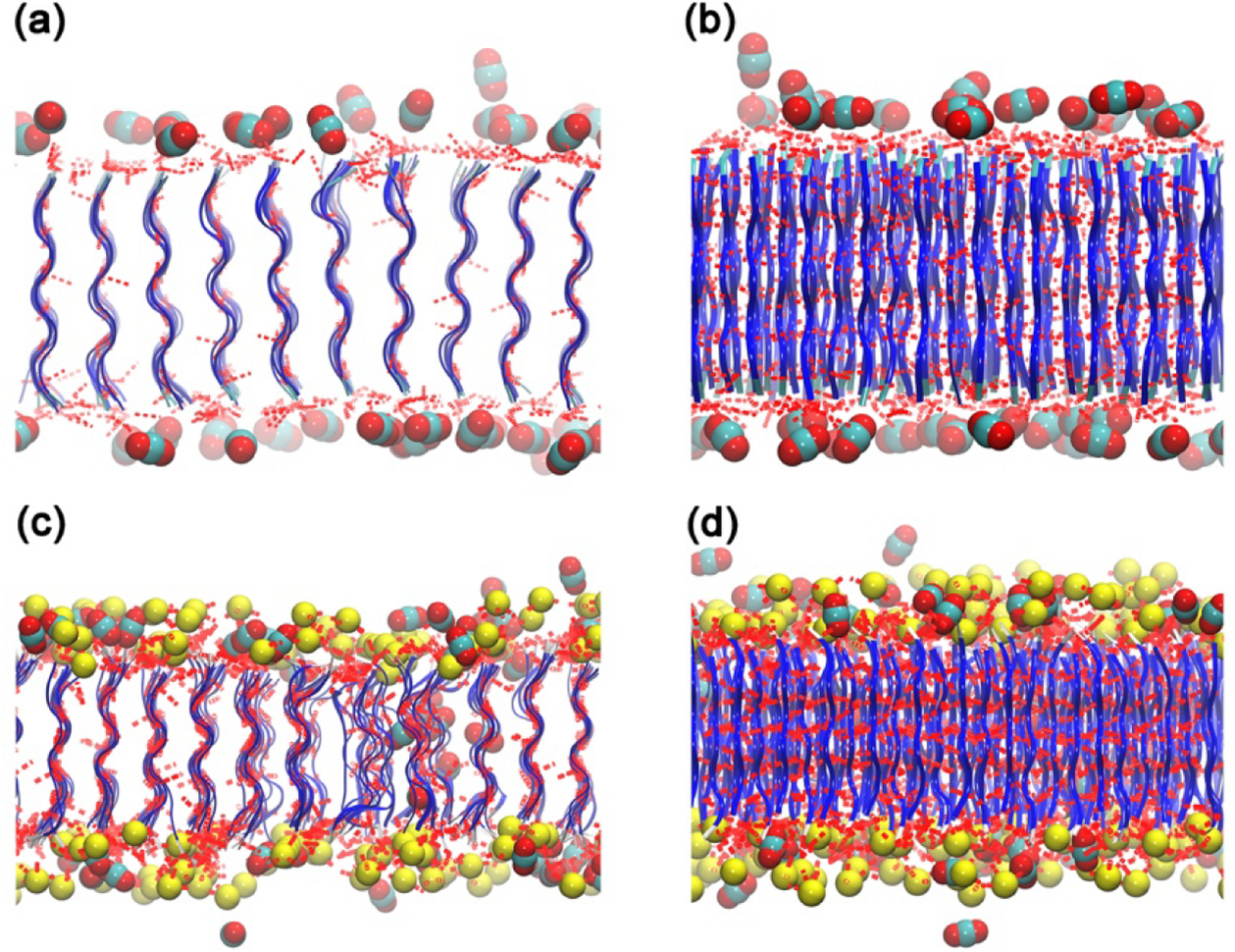
Hydrogen-bond network between CO_2_ and peptide molecules and between peptides in nanomembranes A_6_H and A_6_R after 200 ns of molecular dynamics simulation, shown from two orthogonal orientations: (a, b) A_6_H–CO_2_ and (c, d) A_6_R–CO_2_–Cl^–^ in the zx and zy planes, respectively. Blue ribbons represent peptide backbones, CO_2_ molecules are shown in red (oxygen) and gray (carbon), chloride ions in yellow, and hydrogen bonds as red dashed lines.

### 3.5 Dipole Moment, Kirkwood G-Factor and Einstein’s Diffusion Coefficient

The dipole moment calculations were performed using the trajectory-averaged polarization vectors obtained from the MD simulations of the peptide nanomembranes and their complexes with CO_2_ and chloride ions. The total dipole moment (|M|) was computed from the vector sum of all atomic charges (CHARMM36 parameters) and positions within the simulation box, while its temporal evolution was used to estimate the mean and standard deviation. Complementarily, the Kirkwood G-factor was determined to quantify the degree of orientational correlation between individual molecular dipoles in an infinite system approximation. Together, these parameters provide an assessment of the global and local polarization behavior of the peptide nanomembranes and the influence of CO_2_ adsorption and ionic screening on their electrostatic organization. The results indicate a marked difference in dipolar behavior between the two peptides nanostructure. The isolated A_6_H nanomembrane exhibits a strong permanent dipole moment of 102.7 ± 2.7 D, while the isolated A_6_R nanomembrane displays a significantly lower value (54.8 ± 9.5 D), consistent with a more symmetric charge distribution and partial cancellation of guanidinium dipoles. Upon adsorption of CO_2_, both systems show a decrease in the total dipole magnitude (73.9 ± 46 D for A_6_H–CO_2_ and 23.4 ± 27.8 D for A_6_R–CO_2_–Cl^–^) accompanied by substantially larger fluctuations. This reduction arises from antialignment between the intrinsic dipole of the peptide and the induced polarization of CO_2_ and counterions, which introduces opposite local fields at the interface. The large standard deviations reflect dynamic reorientation of the gas molecules and ions, confirming that the adsorption process occurs under a regime of fluctuating polarization rather than static charge ordering. The Kirkwood G-factors reinforce these observations, showing small absolute values but a consistent increase upon CO_2_ and ion inclusion. For A_6_H system, G rises from 0.00046 (isolated) to 0.00065 (with CO_2_), and for A_6_R system, from 0.00357 to 0.00836 (with CO_2_ and Cl^–^). This trend indicates enhanced local dipole–dipole correlation and partial alignment among neighboring residues and adsorbed species, despite the reduction in the overall dipole magnitude. The increase in G demonstrates that CO_2_ adsorption and ionic structuring promote cooperative polarization at the interface, even as global dipole cancellation occurs across the bilayer. These results confirm that electrostatic screening and molecular dipoles govern the polarization response of the peptide nanomembranes, directly linking interfacial charge distribution to their CO_2_ adsorption mechanisms.

The self-diffusion coefficients (D) of the peptides, CO_2_ molecules, and chloride ions were obtained from the slope of the mean-squared displacement (MSD) according to the Einstein relation, using the linear regime of the MSD curves between 100 and 115 ns from the second simulation window (100–200 ns). This interval corresponds to the fully equilibrated regime, ensuring that diffusional motion is not biased by early-time ballistic or subdiffusive behavior. The resulting values reveal a clear hierarchy in molecular mobility across the systems. For the A_6_H system, the peptide diffusion coefficient is 9.37 ± 3.08 × 10^–9^ cm^2^·s^–1^, reflecting the near-rigid nature of the self-assembled structure, while the CO_2_ molecules exhibit much higher mobility (2.79 ± 0.68 × 10^–4^ cm^2^·s^–1^), consistent with rapid translational dynamics and transient adsorption–desorption events at the membrane interface. These values confirm that the peptide framework remains structurally stable while maintaining a dynamic equilibrium of CO_2_ exchange at its surface. For the A_6_R system, the diffusion coefficient of the peptides has the same order of magnitude as that of A_6_H (5.45 ± 0.13 × 10^–8^ cm^2^·s^–1^), given their comparable supramolecular rigidity and β-sheet packing. The CO_2_ diffusion in this charged environment is considerably higher (5.32 ± 0.09 × 10^–4^ cm^2^·s^–1^), nearly twice the value observed for A_6_H system, indicating that the presence of the ionic layer enhances the mobility of the gas molecules, possibly due to local electrostatic repulsion and reduced residence time near the surface. Conversely, chloride ions display extremely restricted motion (0.54 ± 0.13 × 10^–8^ cm^2^·s^–1^), evidencing strong anchoring to the guanidinium groups of arginine. This combination of high CO_2_ mobility and immobilized counterions reinforces the interpretation of an electrostatically screened interface where ions act as fixed polarization centers while gas molecules diffuse freely above the surface. Together, these results demonstrate the coexistence of structural rigidity, ionic confinement, and gas mobility within the peptide–CO_2_ interfacial systems, confirming that the nanomembranes maintain their supramolecular integrity under dynamic adsorption conditions. Comparing the mobility of CO_2_ in the nanomembranes with its mobility in HEX/MEN,^44^ the reported diffusion coefficient of 2.0 × 10^–3^ cm^2^·s^–1^ indicates a much broader displacement within and around the liquid, where the interactions do not restrict the molecules to a specific interface. In contrast, our results are up to 86% lower, highlighting the behavior in the A_6_H and A_6_R nanomembranes, where CO_2_ remains confined to the membrane interface, as shown in the previous analyses.

At first glance, the higher hydrogen bond (HB) lifetimes observed for the A_6_R membrane together with the increased CO_2_ diffusion coefficient may appear contradictory, as stronger intermolecular interactions are generally associated with reduced mobility. However, these two observables probe different subpopulations of CO_2_ molecules. The HB-lifetime analysis selectively reflects CO_2_ molecules that form persistent, localized interactions with the membrane interface, whereas the mean squared displacement and diffusion coefficients were calculated by averaging over all CO_2_ molecules in the system, including those that are weakly interacting or transiently detached from the surface. In the A_6_R system, a fraction of CO_2_ molecules forms long-lived interactions near guanidinium groups, while a CO_2_’s population remains weakly bound and diffuses more freely, leading to an overall increase in the averaged diffusion coefficient. Therefore, the observed trends in HB-lifetimes and diffusion coefficients are not contradictory but arise from the different molecular populations contributing to each metric.

## 4 Conclusion

The MD simulations performed in this work provided a comprehensive atomistic description of CO_2_ adsorption on bioinspired peptide nanomembranes composed of A_6_H or A_6_R sequences. The results demonstrated that both peptides form stable β-sheet supramolecular assemblies with well-defined bilayer structures that remain intact over 200 ns of simulation. The dominant cohesive forces within these nanomembranes are electrostatic and van der Waals interactions between peptide backbones, ensuring structural integrity under gas exposure. Distinct adsorption regimes emerge depending on the chemistry of the terminal residue: histidine end groups in A_6_H generate localized electrostatic potentials and transient hydrogen bonding with CO_2_, whereas arginine headgroups in A_6_R establish stronger electrostatic coupling mediated by chloride ions. These contrasting behaviors reveal how subtle molecular variations in peptide design control the adsorption energetics and interfacial organization of the gas.

Energetic decomposition analyses confirmed that peptide–peptide interactions are 2 orders of magnitude stronger than peptide–CO_2_ interactions, highlighting that gas adsorption occurs without perturbing membrane cohesion. The A_6_H membrane exhibited the most favorable peptide–CO_2_ Coulombic interaction (≈−6.16 kJ·mol^–1^ per peptide), indicating preferential stabilization of CO_2_ near the histidine-rich surface. In contrast, A_6_R showed partial electrostatic competition between guanidinium groups and chloride counterions, which reduced its effective binding strength but increased the lifetime and cooperativity of HBs. These findings delineate two complementary regimes of CO_2_ capture: a dynamic and reversible process in A_6_H driven by local dipole induction, and a stronger but more rigid adsorption regime in A_6_R governed by ion-mediated electrostatics. Together, these results elucidate how interfacial charge distribution and the ionic microenvironment regulate adsorption strength, reversibility, and selectivity in peptide-based materials.

HBs and polarization analyses further supported these mechanistic interpretations. The A_6_H–CO_2_ system exhibited short-lived hydrogen bonds (≈10 ps) and moderate ΔG values (≈10 kJ·mol^–1^), consistent with transient adsorption at flexible polar sites. Conversely, A_6_R–CO_2_–Cl^–^ displayed markedly longer HBs lifetimes (hundreds of picoseconds) and higher ΔG values (≈18 kJ·mol^–1^), confirming the stabilization effect of the guanidinium–ion framework. Dipole-moment calculations revealed a decrease in global polarization upon gas adsorption, indicating partial antialignment between peptide and CO_2_ dipoles, while Kirkwood G-factors increased with CO_2_ and ion inclusion, reflecting enhanced local cooperativity. These results show that gas adsorption not only modifies interfacial electrostatics but also promotes correlated polarization within the membrane environment, directly linking structure and function at the nanoscale.

Diffusional analyses highlighted the coexistence of structural rigidity and dynamic interfacial transport. CO_2_ molecules displayed diffusion coefficients two to 3 orders of magnitude higher than those of the peptides or ions, confirming rapid surface mobility and reversible adsorption–desorption dynamics. In A_6_R systems, chloride ions were nearly immobilized, forming a fixed electrostatic scaffold that constrained peptide mobility but enhanced CO_2_ motion above the interface. This dual behavior—rigid peptide backbones with mobile adsorbates—demonstrates the adaptive nature of the membranes, where stable structural matrices coexist with dynamic adsorption sites. The simultaneous stability of the membrane and mobility of the gas reinforces the suitability of these systems for long-term, energy-efficient CO_2_ capture operations. Although the present study employs an idealized vacuum-based adsorption model, the mechanistic insights obtained here provide a foundational understanding of peptide–CO_2_ interactions. Future work incorporating multicomponent gas mixtures and explicit solvent environments will be essential to evaluate adsorption performance under realistic operating conditions.

The combined energetic, structural, and dynamic evidence confirms that peptide nanomembranes such as A_6_H and A_6_R constitute promising, sustainable materials for selective CO_2_ capture. Their self-assembled organization provides an intrinsic molecular framework for adsorption without external supports, while their tunable headgroup chemistry allows precise control over binding strength, reversibility, and selectivity. The A_6_H system emerges as an optimal prototype for clean CO_2_ capture, operating through weak yet reversible electrostatic interactions that minimize regeneration energy and environmental impact. These features position peptide-based nanomembranes as viable, biocompatible alternatives to traditional sorbents, offering a pathway toward scalable, low-energy carbon-capture technologies inspired by molecular principles.
